# Supplementary material for: Phototrophic purple bacteria as optoacoustic in vivo reporters of macrophage activity
Source: Nat Commun. 2019 Mar 13;10:1191. doi: 10.1038/s41467-019-09081-5 (PMC6416252; doi:10.1038/s41467-019-09081-5)
Supplement: Supplementary file 1 — Supplementary Information [file 41467_2019_9081_MOESM1_ESM.pdf]

Supplementary Information

Phototrophic purple bacteria as optoacoustic *in vivo* reporters of macrophage activity

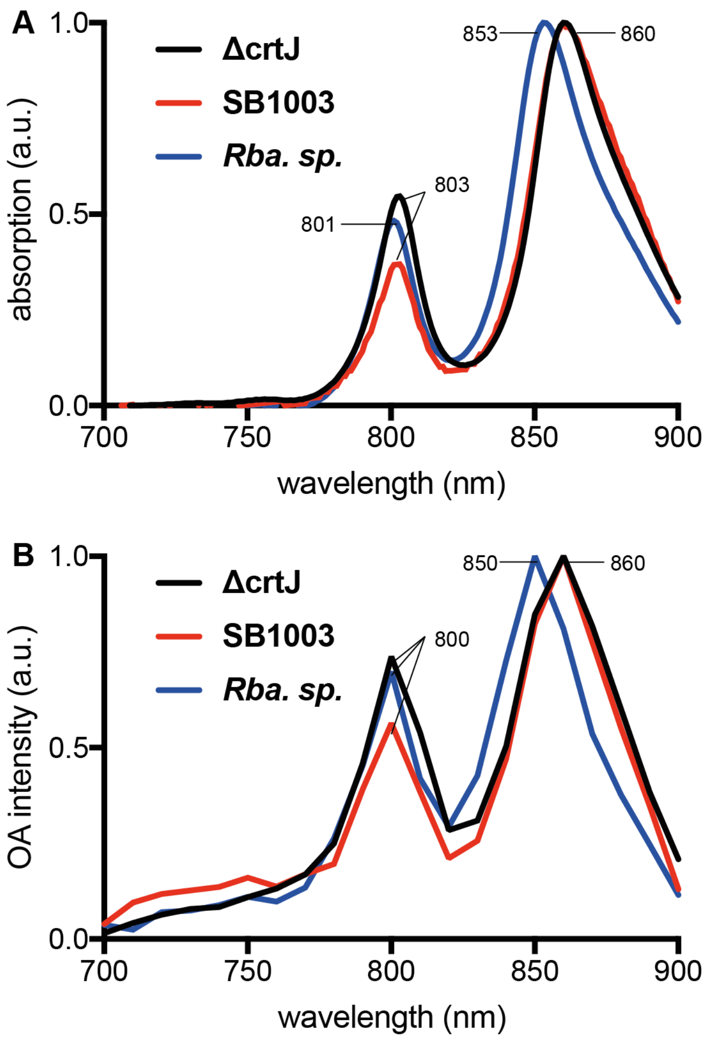

Supplementary Figure 1: Whole cell absorption spectra (A) and *in vitro* OA spectra (B) of *Rba. capsulatus* SB1003/Δ*crtJ* (ΔcrtJ), SB1003 and *Rba. sphaeroides* (*Rh. sp.*). Spectra are normalized to the maximum between 700 and 900 nm with wavelength of maxima indicated next to the peaks.

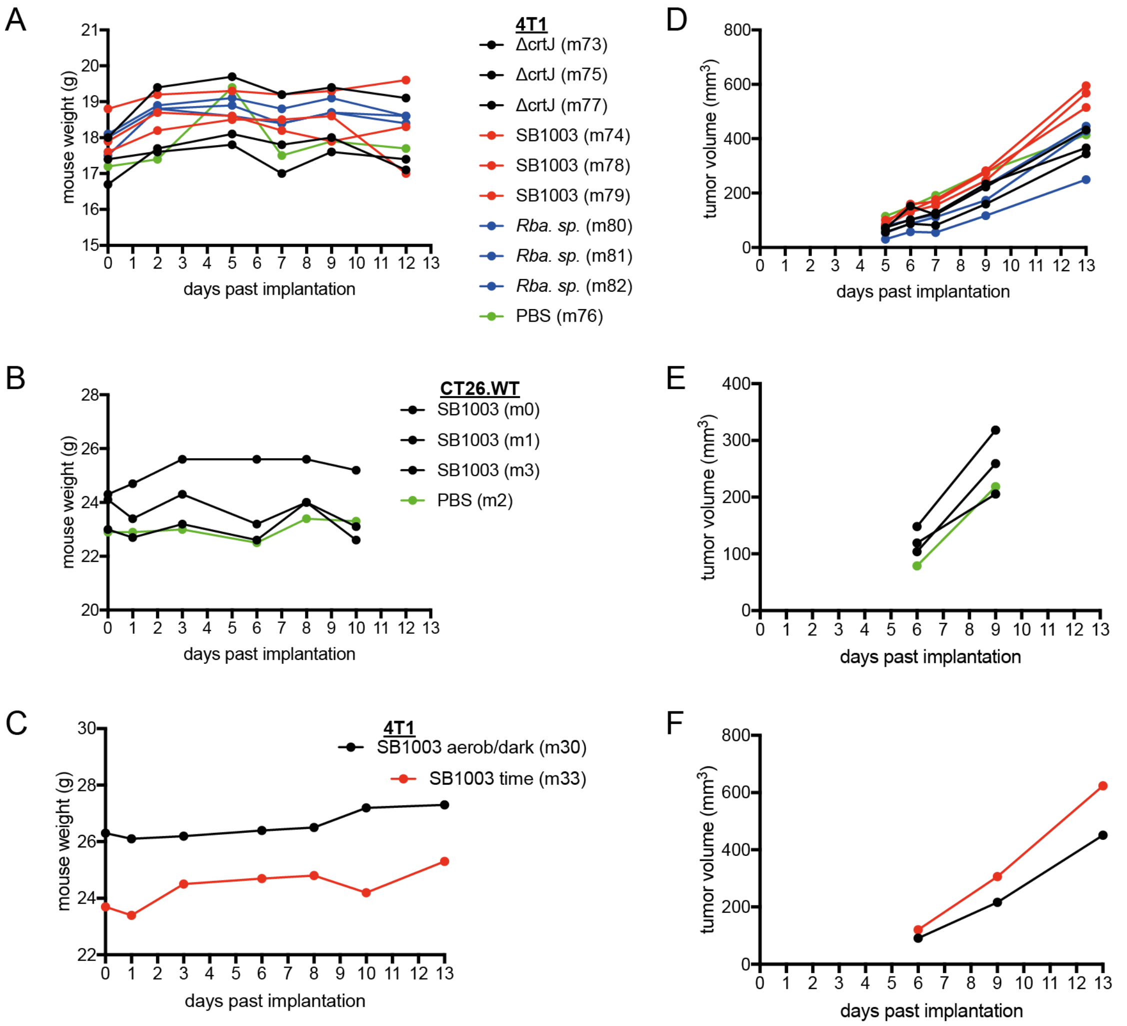

Supplementary Figure 2: Weight (A-C) and tumor size (D-F) of mice used in the experiments. The days of injection for the different samples are indicated as color bars under the horizontal axis. Color scheme as depicted in the legend. (**A and D**) 4T1 tumors injected with *Rba. capsulatus* SB1003 *ΔcrtJ*, *Rba. capsulatus* SB1003 and *Rba. sphaeroides* as well as PBS as a control. (**B and E**) CT26.WT injected with *Rba. capsulatus* SB1003. (**C and F**) 4T1 tumors injected with *Rba. capsulatus* SB1003 grown aerobe and in the dark as well as the sample that was only measured at the beginning and at the end.

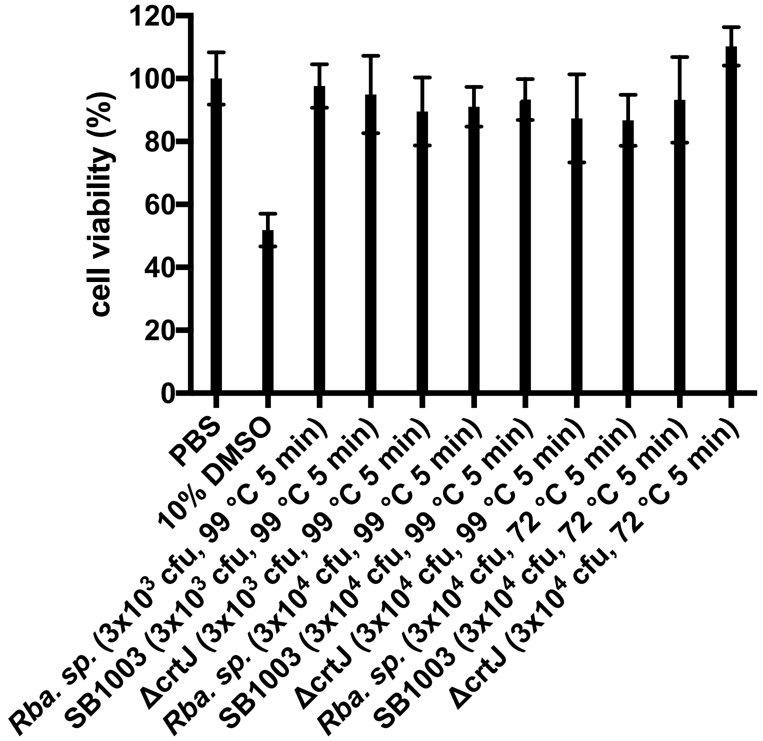

Supplementary Figure 3: Effects of heat-inactivated *Rhodobacter* cells on viability of 4T1 cells (MTT assay). The assay was performed as described in the methods section. Data is normalized to the PBS control (=100 %). 10 % DMSO was used as a positive control (inducing cell death). Average and error bars (standard deviation) are shown for n=8.

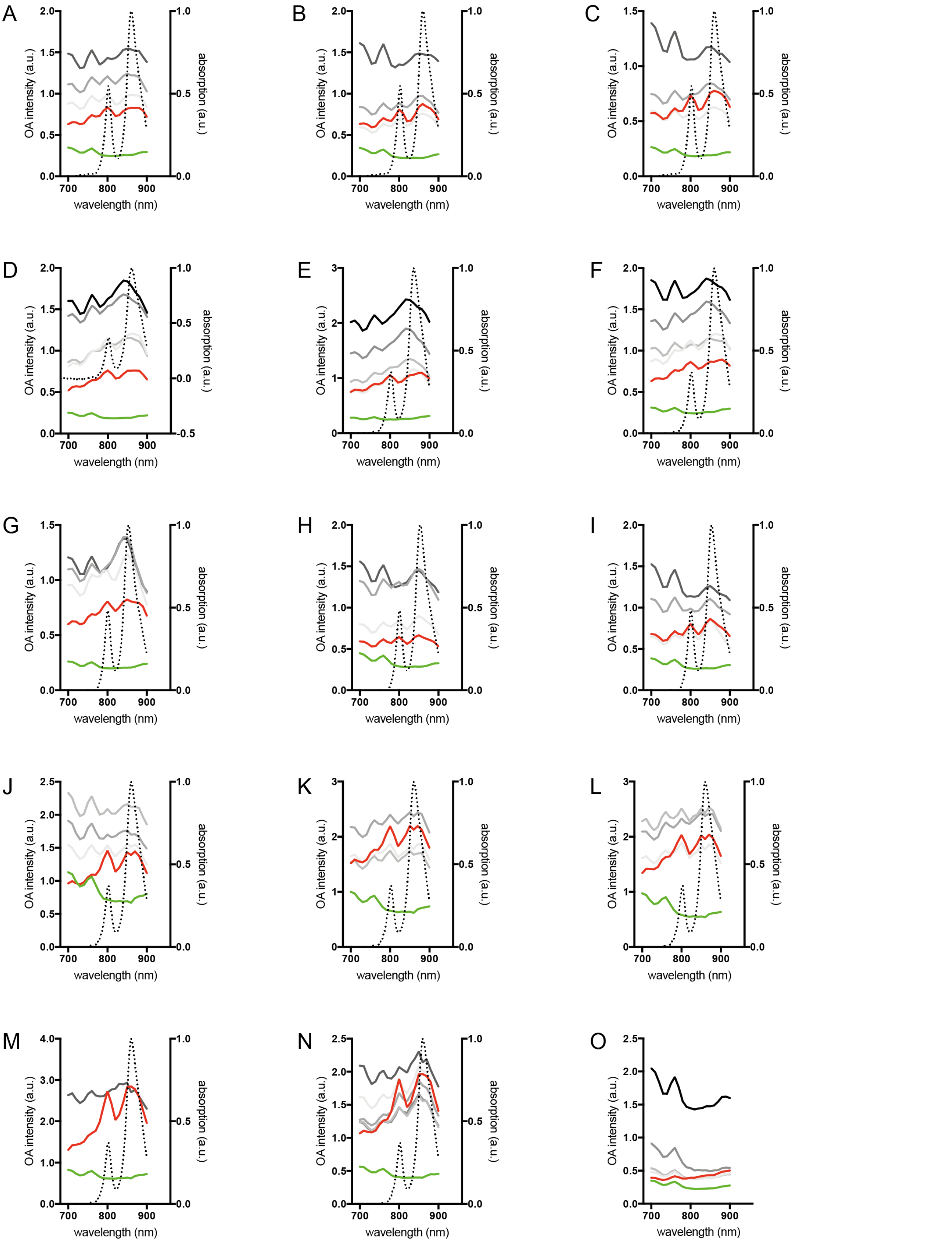

Supplementary Figure 4: Optoacoustic and absorption spectra of tumor ROI for all mice. Coloring as in Figure 1 with spectra before injection in green, spectra immediately after injection in red (t = 0 h) and spectra of subsequent timepoints in shades of grey (t = 24, 48/72/96, 168/192 h). The absorption spectra of the respective injected bacteria are shown as dotted lines. Three mice each: 4T1 tumors injected with *Rba. capsulatus* SB1003 *ΔcrtJ* (**A-C**), *Rba. capsulatus* SB1003 (**D-F**) and *Rba. sphaeroides* (**G-I**) as well as mice with CT26.WT tumors injected with *Rba. capsulatus* SB1003 (**J-L**). **M** shows the spectra for *Rba. capsulatus* SB1003 measured only at two timepoints after injection while **N** depicts *Rba. capsulatus* SB1003 grown microaerobically in the dark. **O** shows the spectral development of a control mouse injected with PBS. Scans

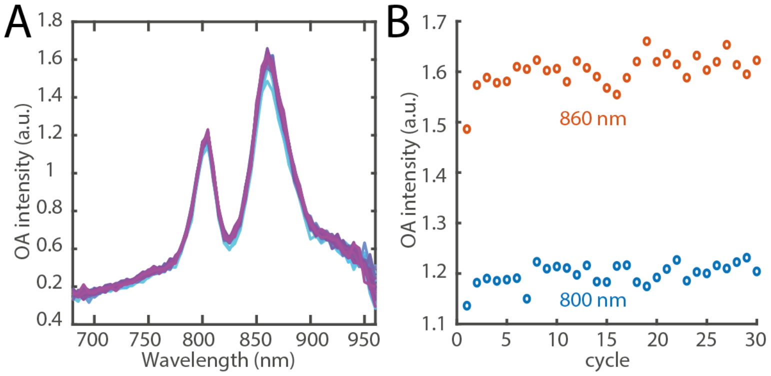

Supplementary Figure 5: Bleaching of live *Rba. capsulatus* SB1003 under MSOT imaging conditions. A: 30 consecutive optoacoustic spectra of *Rba. capsulatus* SB1003 immobilized in low melting agarose accounting for 15 min of imaging. **B:** 800 nm and 860 nm maxima of the spectra shown in A.

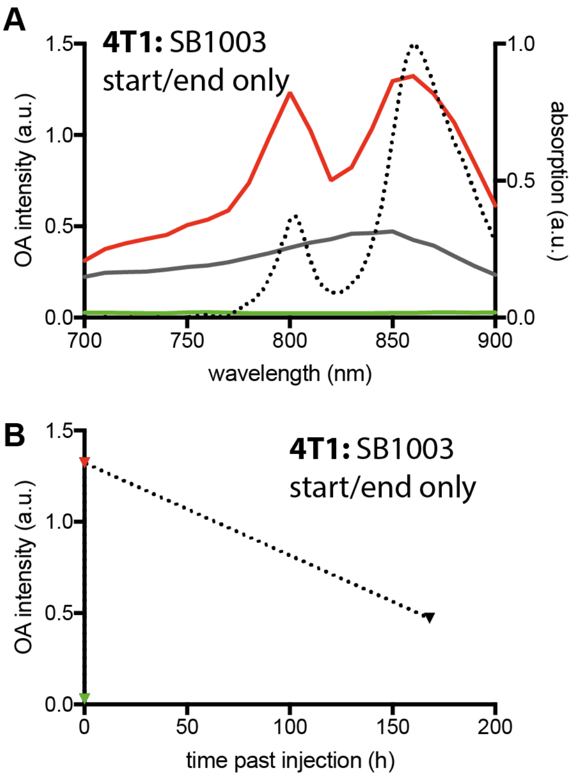

Supplementary Figure 6: Control mice injected with *Rba. capsulatus* SB1003 measured only at first and last time point (A and B). To exclude confounding effects of the laser light used to elicit OA signals we injected a mouse with *Rba. capsulatus* SB1003 grown under standard conditions and recorded the first and last time point only (**A** and **B**). The changing peak signature can also be observed in this case confirming that the measurement conditions have no influence on the peak change. Visualization and coloring as in Figure 1.

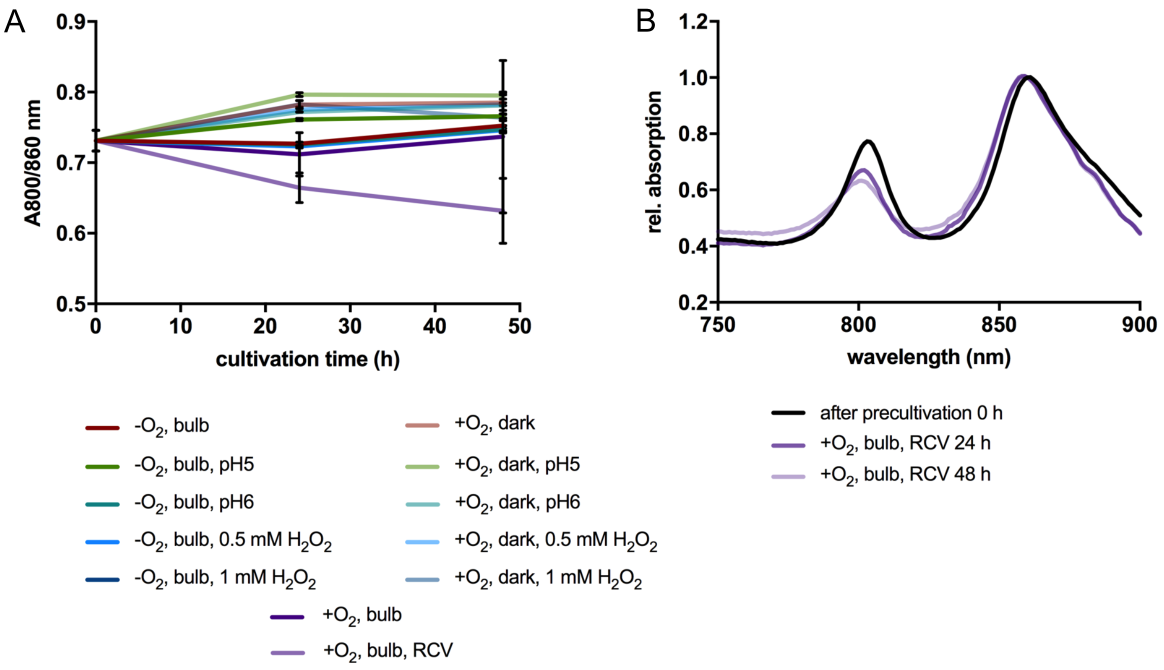

Supplementary Figure 7: Influence of shifted cultivation conditions on *R. capsulatus* BChl *a* absorption peaks. **(A)** *Rba. capsulatus* SB1003 was cultivated under anaerobic phototrophic conditions using IR light for illumination in RCV medium for 3 days to gain sufficient cell material in the same growth state (cultivation in several hungate tubes in parallel). These cells were used to inoculate new cultures in RCV 2/3 PY medium (in hungate tubes) with a starting cell density of $OD_{660\ nm}$ = 0.05. After 24 h of cultivation under IR light cells were exposed to different conditions by starting test cultures with a cell density of $OD_{660\ nm}$ = 0.5 in different setups before measuring absorbance spectra of whole cells to monitor the BChl *a* signature and calculate the ratio of the absorption at 800 nm and 860 nm.

In detail: Test cultures of bacteria were grown in RCV 2/3 PY medium in hungate tubes under anaerobic phototrophic conditions under bulb light as a control (-O_2, bulb). The same setup was implemented with RCV 2/3 PY medium buffered to pH 5.0 or pH 6.0 (-O_2, bulb, pH5 and pH6), or supplemented with H_2O_2 (final concentrations of 0.5 mM or 1 mM; -O_2, bulb, 0.5 mM H_2O_2 and 1 mM H_2O_2). In addition, microaerobic conditions were implemented in 100-ml Erlenmeyer flasks containing 50 ml RCV 2/3 PY medium (+O_2, dark), medium buffered to pH5 or pH6 (+O_2, dark, pH5 and pH6), or supplemented with 0.5 mM or 1 mM H_2O_2 (+O_2, dark, 0.5 mM H_2O_2 and 1 mM H_2O_2), and cells were incubated at constant shaking frequencies (130 rpm) in the dark. Finally, microaerobic cultures were incubated shaking under bulb light in Erlenmeyer flasks containing 50 ml RCV 2/3 PY medium (+O_2, bulb) or RCV minimal medium (+O_2, bulb, RCV). All cultivations were conducted in triplicates. Whole cell absorbance spectra of 100 µl samples were recorded using a TECAN Infinite plate reader immediately after the pre-cultivation, i.e. at 0 h, and at 24 h and 48 h of cultivation after the implementation of changed conditions. The absorption at 800 nm was monitored relative to the absorption at 860 nm (A 800 nm/860 nm). All data represent the average and standard deviation of three independent measurements.

(B) Mean absorbance spectra of cells incubated under the latter conditions for 24 h and 48 h are shown in comparison to spectra recorded immediately after the precultivation (after precultivation 0 h). Data was normalized as relative absorbance to the absorption maximum of BChl *a* at 860 nm.

A

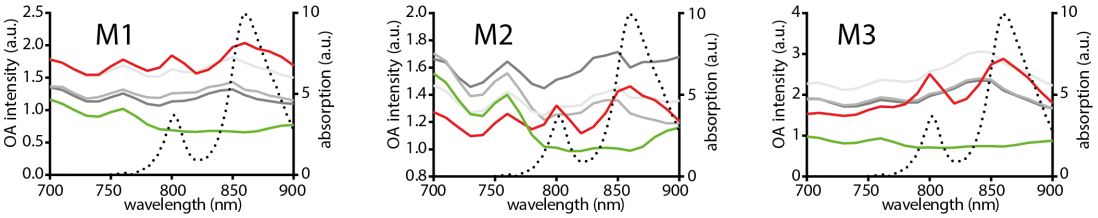

Supplementary Figure 8: Control mice injected with *Rba. capsulatus* SB1003 that have been lysed via French press treatment. A: Optoacoustic mean spectra of unmixed pixels of three mice injected with lysed bacteria. Green denotes spectra before injection, red immediately after injection and shades of grey timepoints 24, 48 and 72 h past injection. The absorption spectra of *Rba. capsulatus* SB1003 are shown as dotted lines.

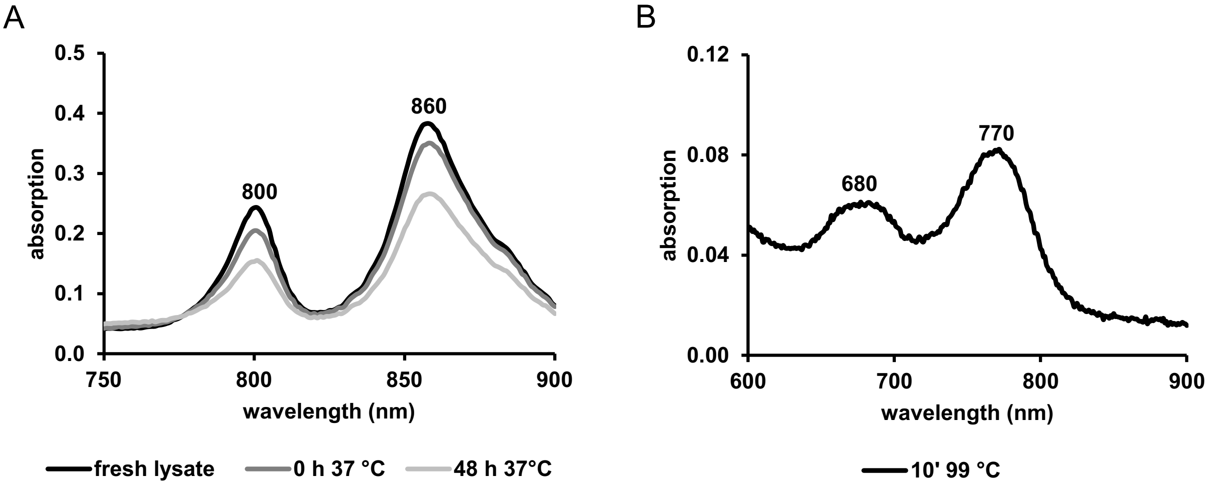

Supplementary Figure 9: Absorption spectra of *R. capsulatus* French Press cell lysates during incubation at different temperatures. *Rba. capsulatus* SB1003 cells were cultivated and lysates were prepared exactly as the samples that were injected into mice: *Rba. capsulatus* was pre-cultivated under anaerobic phototrophic conditions using IR light for illumination in RCV medium for three days. These cells were used to inoculate ten cultures in hungate tubes with RCV 2/3 PY medium in parallel with a starting cell density of $OD_{660\ nm}$ = 0.05. After 24 h of cultivation under IR light, cell material corresponding to $OD_{660\ nm}$ = 400 was pelleted and re-suspended in 20 ml PBS, and subjected to disruption in a French Press apparatus (500 bar). The cell suspension was passed through the apparatus eight times to ensure complete lysis, which was confirmed by plating samples on agar plates (i.e. no colony formation). Absorption spectra of the fresh lysate were recorded (fresh lysate), before samples were stored at -80 °C over night. These samples were used to assess the effect of specific temperatures on the absorption spectrum.
(A) Lysate samples were incubated for 48 h at 37 °C, i.e. the temperature the bacterial samples are exposed to in mice experiments (0 h and 48 h). While the signal intensity decreased over time under these conditions, the absorption maxima remained unchanged at 800 nm and 860 nm, as indicated. The same trend was observed also for prolonged incubation.
(B) After 48 h incubation at 37 °C, lysate samples were heat-denatured (99 °C for 10 minutes), before another spectrum was recorded. The signal intensity was strongly decreased and the absorption maxima were blue-shifted to 680 nm and 770 nm. Absorbance spectra were recorded using a TECAN Infinite plate reader and sample volumes of 100 µl.

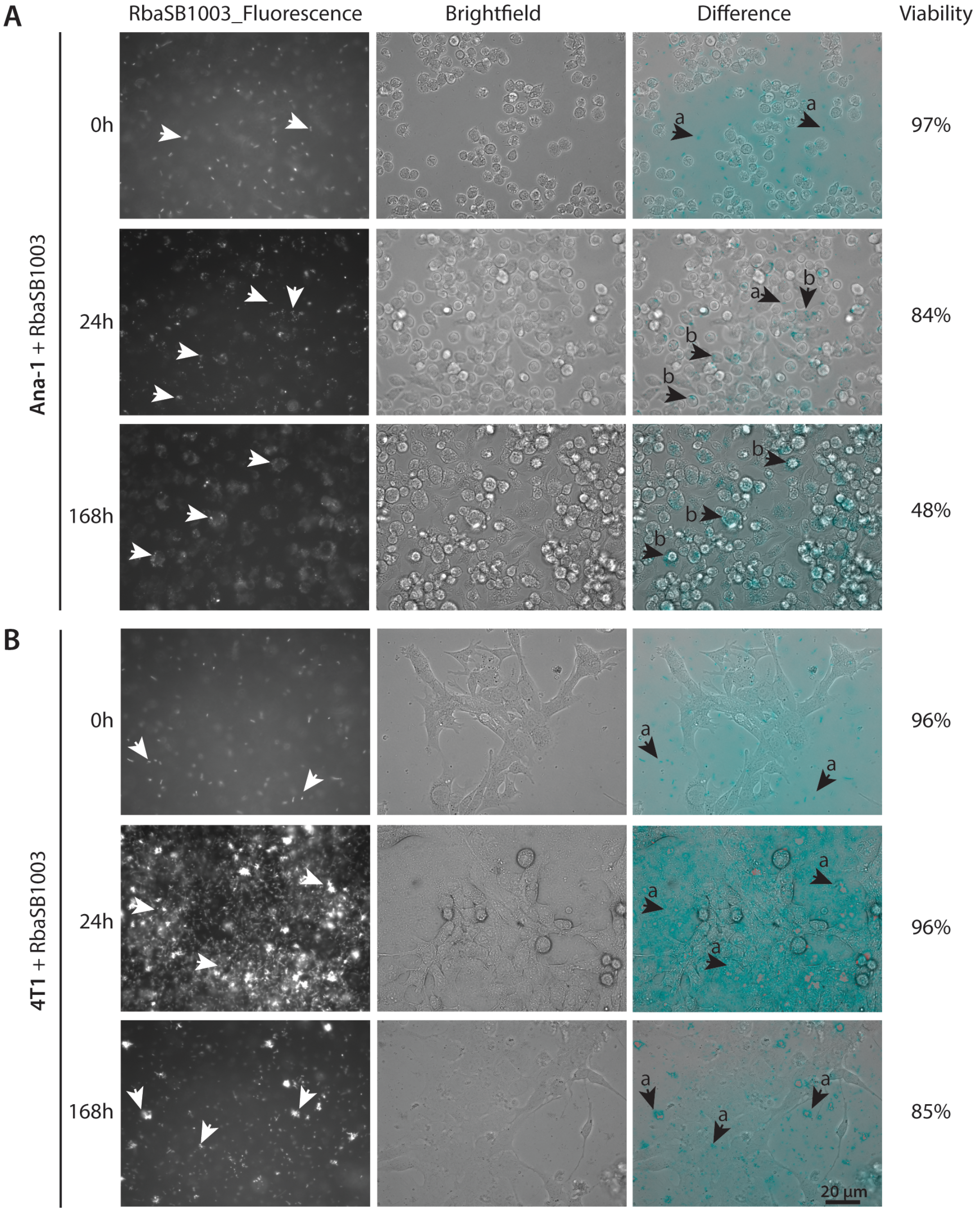

Supplementary Figure 10: Live cell imaging of Ana-1 macrophages (A) and 4T1 tumor cells (B) in the presence of *Rba. capsulatus* SB1003 over time. Immediately after *Rhodobacter* addition to cells at a ratio of 50:1, fluorescent bacteria are detected via mCherry-filter throughout the media in both Ana-1 and 4T1 cultures indicated by arrow a (0 h). While the majority of free-living bacteria (arrow a) is ingested by Ana-1 within 24 h and remains intracellularly for up to 168 h (arrow b), 4T1 tumor cells do not endocytose *Rhodobacter* at any detectable amount. Interestingly, the wavelength of fluorescent signal of intracellular *Rhodobacter* in Ana-1 after 168 h shifted from mCherry to Cy7. Viability scoring (right of each panel) at each time point shows macrophage sensitivity towards *Rhodobacter* post ingestion and prolonged exposure. In contrast, 4T1 cells show no decreased viability caused specifically by the presence of *Rhodobacter* but rather by extended cultivation. It must be noted that the imperfect colocalization of the fluorescent - and brightfield signals of extracellular bacteria is due to their motility and a 2-second exposure time to capture their fluorescence. Corresponding images for all strains at all timepoints have been recorded with the same settings. Scale bar is 20 µm.

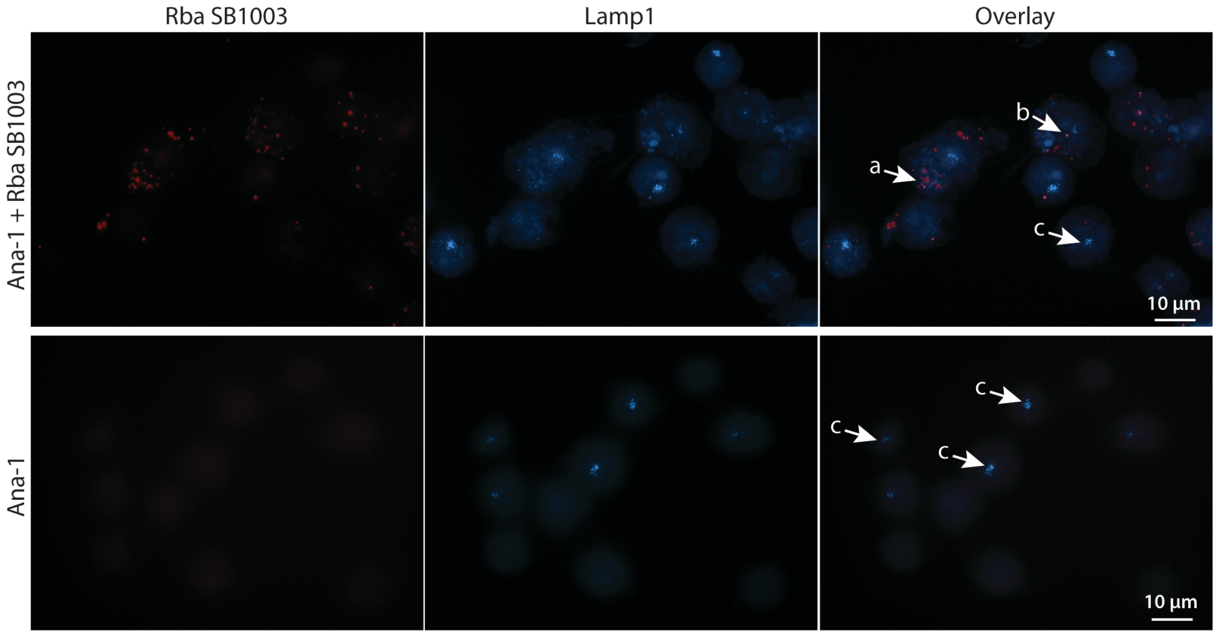

Supplementary Figure 11: Lysosomal staining of Ana-1 after treatment with *Rba. capsulatus* SB1003. Ana-1 macrophages were grown on cover glasses in the presence and absence of *Rhodobacter* cells for 24 h as described in Supplementary Figure 10A prior to paraformaldehyde-fixation and immunofluorescence staining with anti-Lamp1 antibody (Alexa-405). Minimal (arrow b) to no colocalization (arrow a) of internalized bacteria fluorescence (red, Cy7) with lysosomes/late endosomes (blue, arrow c) was detected across the population of macrophages. Scale bar is 10 µm.

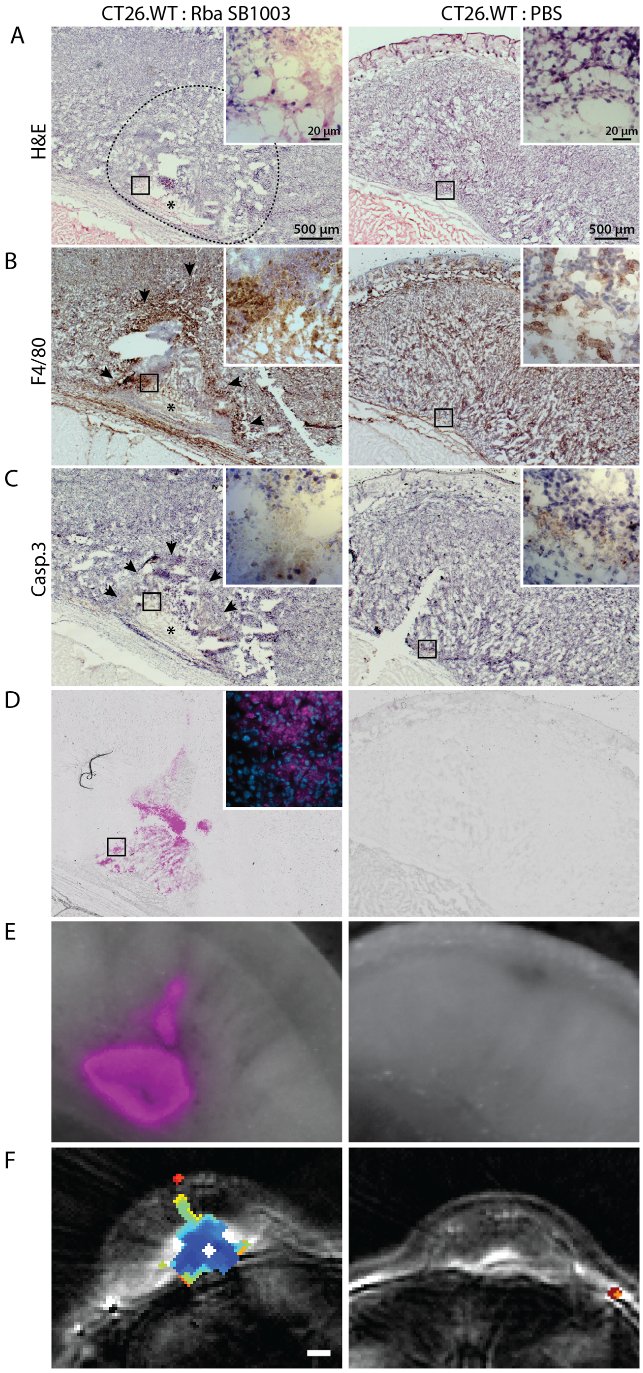

Supplementary Figure 12: Histology and OA signature of CT26.WT tumors injected with *Rba. capsulatus* SB1003 or PBS. Consecutive tumor sections of 10 µm thickness **(A-D)** show hematoxylin and eosin stain (H&E) **(A)**, immunohistochemistry with macrophage marker F4/80 **(B)** or apoptosis marker cleaved caspase 3 **(C)** both brown while counterstained with blue hematoxylin. Ingested and disintegrated Rhodobacter are detected via their fluorescent signal **(D)** (magenta, Cy7, Dapi is shown in magnification only) and localize to the area of strong macrophage accumulation and apoptosis. Tumor halves **(E)** before sectioning and IHC show strong Rhodobacter fluorescence (excitation: 740/40 nm bandpass, emission: 850 nm longpass). Boxes in the main images (2.5 x magnification, scale bar 500 µm) indicate areas shown in upper right insets (40 x magnification, scale bar 20 µm). The semicircle in the top image indicates the region primarily influenced by Rhodobacter injection with the asterisk in the region of massive cellular destruction, presumably the position of the initial bacterial bolus. Arrows indicate transition zones between natural tumor tissue and zones where macrophages ingested Rhodobacter. Those areas show strong fluorescence suggesting the same chromophores as primary source of OA signal **(F)**. The PBS control injection had a minor influence on the morphology of the tumor with few apoptotic cells in the injection area at the base of the tumor and a homogenous distribution of macrophages throughout the tissue.

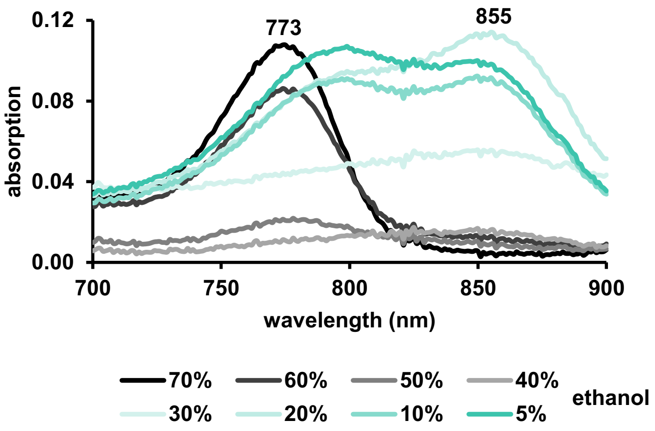

Supplementary Figure 13: Absorption spectra of BChl *a* in different solvent compositions. Bacteriochlorophyll *a* (BChl *a*) was obtained from Sigma Aldrich (CAS 17499-98-8; B5906 Sigma-Aldrich). A stock solution (2 mg/ml) was prepared in ethanol (p.a.), from which 5 µl samples were used to complete different mixtures of water and ethanol with final ethanol concentrations ranging from 70% to 5%. Absorbance spectra were recorded using a TECAN Infinite plate reader and sample volumes of 100 µl. While BChl *a* showed a typical maximum of the monomer in ethanol as solvent at ca. 773 nm (70% and 60%), intermediate ethanol concentrations led to a loss of specific signals (50%, 40% and 30%). In solutions with lower ethanol concentrations (20%, 10% and 5%), however, an absorption around 800 nm and 855 nm were detected, with the most prominent shift to 855 nm at 20% ethanol.

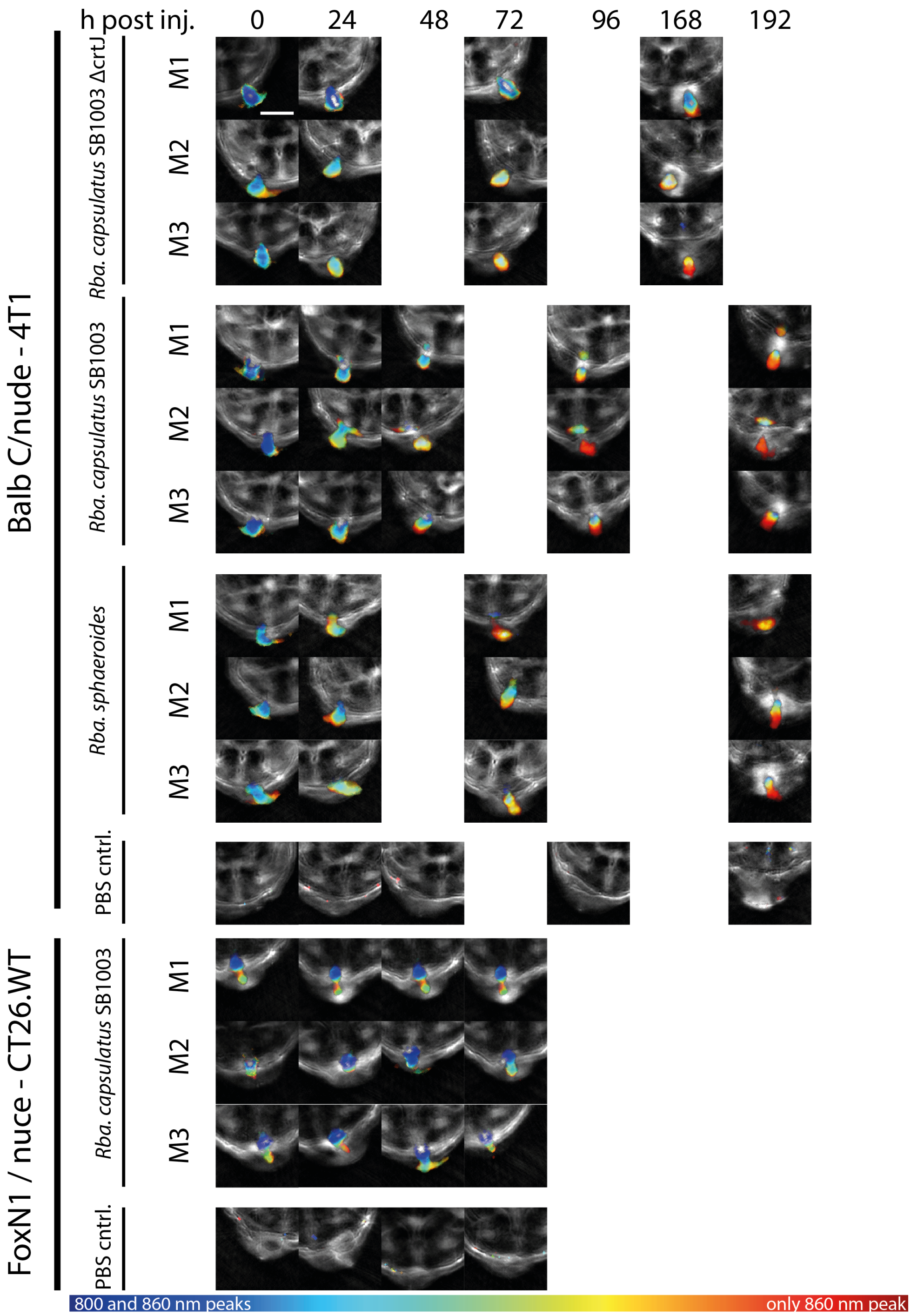

h post inj.
0
24
48
72
96
168
192
Balb C/nude - 4T1
Rba. capsulatus SB1003 ΔcrtJ
Rba. capsulatus SB1003
Rba. sphaeroides
PBS cntrl.
FoxN1 / nuce - CT26.WT
M1
M2
M3
800 and 860 nm peaks
only 860 nm peak

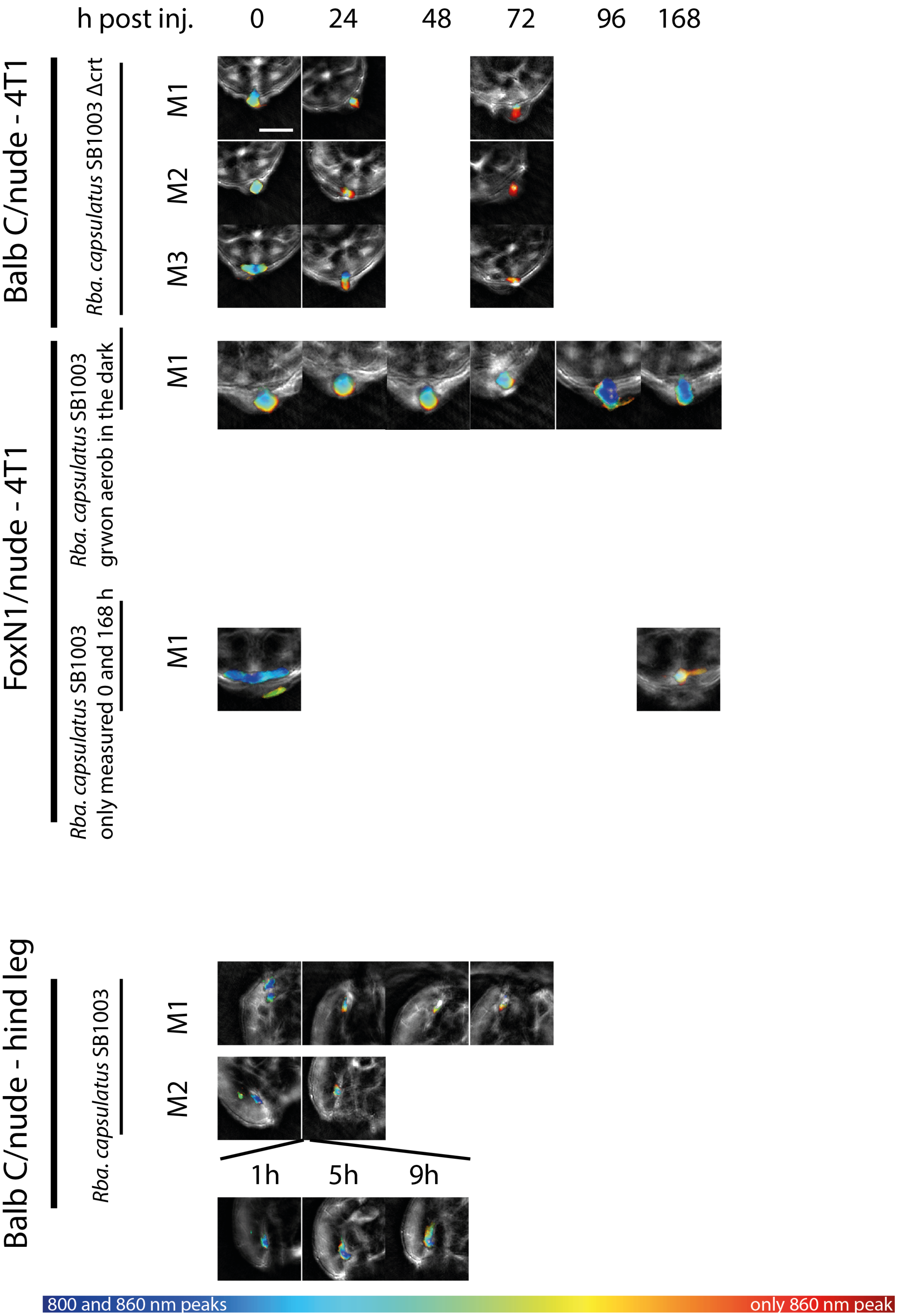

Supplementary Figure 14: Spectral analysis of the unmixed pixels in the tumor region for all mice at all timepoints. Shown are maximum intensity projections. Color coding describes the spectral signature, ranging from blue for equally high 800 and 860 nm peaks to red for 860 nm peak only. Scale bar is 5 mm in all images.

Supplementary Table. 1: Accumulation of BChl *a* in different phototorphic α proteobacteria under different growth conditions. Since efficient photopigment synthesis is favored by lowered oxygen accessibility, we analyzed BChl *a* absorption and cell growth at anaerobic and microaerobic conditions in different minimal media (RCV, SISTROM and CENMED), complex media (PY) as well as in a mixture of complex PY medium and RCV medium. For anaerobic growth different light sources were used for illumination as specified in the Methods section. For a better comparability, BChl *a* absorption at λ=860 nm was normalized to the respective cell density determined as optical density at 660 nm (A 860 nm/660 nm). B10S exhibited a less robust growth behavior at 37°C and serious growth impairment in minimal medium under anaerobic conditions. All data represent the average and standard deviation of three independent measurements. n.g.: no growth.

strain	media	microaerobic growth	- O_2 / IR	- O_2 / BL	- O_2 / IR and BL	- O_2 / light bulbs
Rba. capsulatus	minimal	0.62 +/- 0.13	n.g.	1.22 +/- 0.19	1.83 +/- 0.02	1.46 +/- 0.12
**B10S**	mixture	1.37 +/- 0.03	2.33 +/- 0.13	2.47 +/- 0.06	2.07 +/- 0.09	2.05 +/- 0.09
	complex	0.97 +/- 0.05	1.89 +/- 0.03	2.37 +/- 0.12	1.57 +/- 0.42	1.49 +/- 0.27
Rba. capsulatus	minimal	0.95 +/- 0.14	1.57 +/- 0.24	1.00 +/- 0.01	1.67 +/- 0.05	1.43 +/- 0.04
**SB1003**	mixture	1.41 +/- 0.04	2.15 +/- 0.04	1.70 +/- 0.17	2.07 +/- 0.02	1.86 +/- 0.02
	complex	0.79 +/- 0.01	1.90 +/- 0.04	1.58 +/- 0.11	2.00 +/- 0.07	1.40 +/- 0.04
Rba. capsulatus	minimal	1.12 +/- 0.04	1.83 +/- 0.05	2.16 +/- 0.06	1.74 +/- 0.08	1.54 +/- 0.07
**SB1003Δ*crtJ***	mixture	1.45 +/- 0.08	2.48 +/- 0.05	2.51 +/- 0.09	2.29 +/- 0.03	2.16 +/- 0.04
	complex	0.60 +/- 0.05	2.15 +/- 0.09	2.20 +/- 0.07	1.97 +/- 0.03	1.39 +/- 0.01
Rba. sphaeroides	minimal	0.91 +/- 0.02	1.19 +/- 0.07	1.20 +/- 0.08	1.02 +/- 0.02	0.98 +/- 0. 02
**ATH 2.4.1**	mixture	0.87 +/- 0.05	1.61 +/- 0.09	1.96 +/- 0.04	1.37 +/- 0.07	1.44 +/- 0.02
	complex	0.62 +/- 0.04	1.66 +/- 0.03	2.41 +/- 0.51	1.37 +/- 0.07	1.19 +/- 0.11
Rsp. centenum	minimal	1.10 +/- 0.01	0.95 +/- 0.04	n.g.	1.11 +/- 0.03	0.96 +/- 0.26
**DSMNo: 8284**	mixture	0.95 +/- 0.04	n.g.	n.g.	n.g.	n.g.
	complex	0.90 +/- 0.09	n.g.	n.g.	n.g.	n.g.

Supplementary Table 2: Mice imaged in this study. Crtj = *Rba. capsulatus* SB1003 ΔcrtJ, sb1003 = *Rba. capsulatus* SB1003, rba = *Rba. spaeroides,* sb1003 french press = *Rba. capsulatus* SB1003 lysed by French press treatment. The numbers at the timepoints are internal scan numbers.

number in group	mouse model	tumor / model	injection	pre	0 h post inj.	24 h	48 h	72 h	96 h	168 h	192 h
1	balb/c nude	4t1	crtj	14	15	43		54		64	
2	balb/c nude	4t1	crtj	33	34	46		56		66	
3	balb/c nude	4t1	crtj	35	36	48		58		68	
1	balb/c nude	4t1	sb1003	2	6	10	44		55		65
2	balb/c nude	4t1	sb1003	4	7	12	49		59		69
3	balb/c nude	4t1	sb1003	5	8	13	50		60		70
1	balb/c nude	4t1	rba	37	38	51		61			71
2	balb/c nude	4t1	rba	39	40	52		62			72
3	balb/c nude	4t1	rba	41	42	53		63			73
	balb/c nude	4t1	PBS	3	9	11	47		57		67
3	FoxN1 Nude	ct26	sb1003	16	17	31	41	51			
2	FoxN1 Nude	ct26	sb1003	18	19	32	42	49			
1	FoxN1 Nude	ct26	sb1003	20	21	33	43	50			
	FoxN1 Nude	ct26	PBS	11	12	30	40	52			
1	balb/c nude	4t1	sb1003, french press	7	8	15		23			
2	balb/c nude	4t1	sb1003, french press	5	6	16		21			
3	balb/c nude	4t1	sb1003, french press	3	4	17		22			
	FoxN1 Nude	4t1 (first / last)	sb1003	3	4					56	
	balb/c nude	muscle	sb1003	2	3	4	5	7			
				**pre**	**0 h post inj.**	**1 h**	**5 h**	**9 h**	**24 h**		
	balb/c nude	muscle	sb1003	1	2	3	4	5	6		

Supplementary Note 1: Potential confounders of the spectral signature

Each imaging volume (voxel) recorded in OA tomography imaging represents the OA signal emitted by all absorbers excited at the given wavelength; be it endogenous absorbers like blood or lipids or exogenous agents like in our case bacteria with a strong absorbance at 800/860 nm. Consequently, the extent to which the pure agent spectrum is affected by endogenous absorbers depends on the wavelength as well as the concentrations of the confounding absorbers (Supplementary Figure 15). Accordingly, the bacterial spectra generated by MSOT could be differently perturbed depending on the immediate tumor surrounding. With this in mind we elucidated if the interplay between confounding absorbers and bacterial agent are able to mimic the spatiotemporal spectral changes we observe. To this end we simulated additive convolutions of the known bacterial spectrum with 800/860 nm peaks (*Rba. capsulatus* SB1003 absorption spectra) with "differed amounts" of oxygenated and deoxygenated hemoglobin as well as lipids and compared these convoluted spectra with a MSOT spectra exhibiting solely an 860 nm peak, regularly observed at later time points in our experiments (Supplementary Figure 16). The simulated spectra with the highest correlation is the source spectrum of *Rba. capsulatus* SB1003 without any additional confounding absorbers. This suggests that no combination of bacterial agent spectra and endogenous absorber spectra can mimic the observed change of spectral signature we observe. Secondly, we studied if additive imprecisions of the MSOT device in the determination of the agent spectra (i.e. peak shifts by several nm) can result in the observed spectral change. We simulated additive convoluted spectra of the source spectrum of *Rba. capsulatus* SB1003 that were randomly shifted by +/- 0 20 nm (2 spectral scanning steps, Supplementary Figure 17 B), by +/- 0-50 nm (5 steps, Supplementary Figure 17 C) or a combination of both (Supplementary Figure 17 D). For a spectral offset of 20 nm this has almost no effect on the spectra. However, offsets of up to 50 nm can possibly reproduce the broadened 860 nm-only peak spectra we interpreted as biological driven. Importantly, however, this simulated single peak is generally shifted by 30 – 50 nm to the red while the peak we observe experimentally is blue shifted. Secondly, if peak substructure persists in the convolutions it does not match with the experimentally observed peak substructure of peaks intermediate between the 800/860 nm and the 860 nm-only peaks. Thirdly, the spectral uncertainty as interpreted from the maxima of all unmixed pixels in our experiments is mostly around 10 nm (Supplementary Figure 18) suggesting that a scenario with up to 50 nm offsets is unlikely. Lastly, the measured spectra are the convolution of the physical characteristics of absorbers in the voxel, meaning that the spectral uncertainty can only arise on the level of the measurement (the final convolution). This makes the described scenario of additive peak uncertainties rather unrealistic.

In summary, we think that the observed spectral changes are largely caused by changes in the bacteria and their tumor surrounding. However, it has to be noted that due to the described methodological effects spectral signatures of individual pixels may still be perturbed. Due to this, changes should not be interpreted numerically on a per-pixel-basis but rather phenomenological on a regional basis.

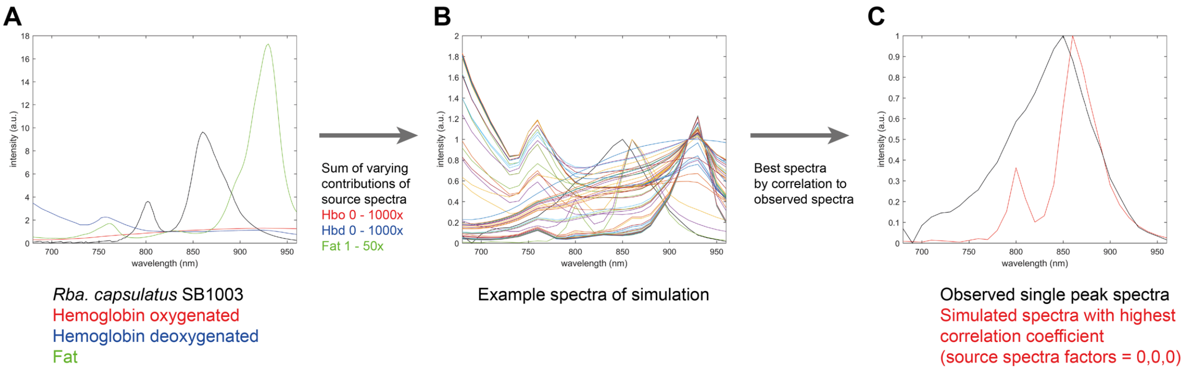

Supplementary Figure 15: Simulation of spectral convolution of *Rhodobacter* peaks (*Rba. capsulatus* SB1003) with major endogenous absorbers in the spectral window (oxygenated and deoxygenated hemoglobin and lipids). For the simulation normalized spectra of all potentially confounding absorbers and *Rba. capsulatus* SB1003 have been summed (**A**). To simulate different quantities of the absorbers spectra have been multiplied by different factors for each simulated spectrum (**B**). In total 2525541 different convolution spectra have been calculated. For each spectrum the correlation to a representative experimentally observed single peak spectrum has been calculated. The convoluted spectrum with the highest correlation coefficient (**C**) is the source spectrum of *Rba. capsulatus* SB1003 without any confounder added.

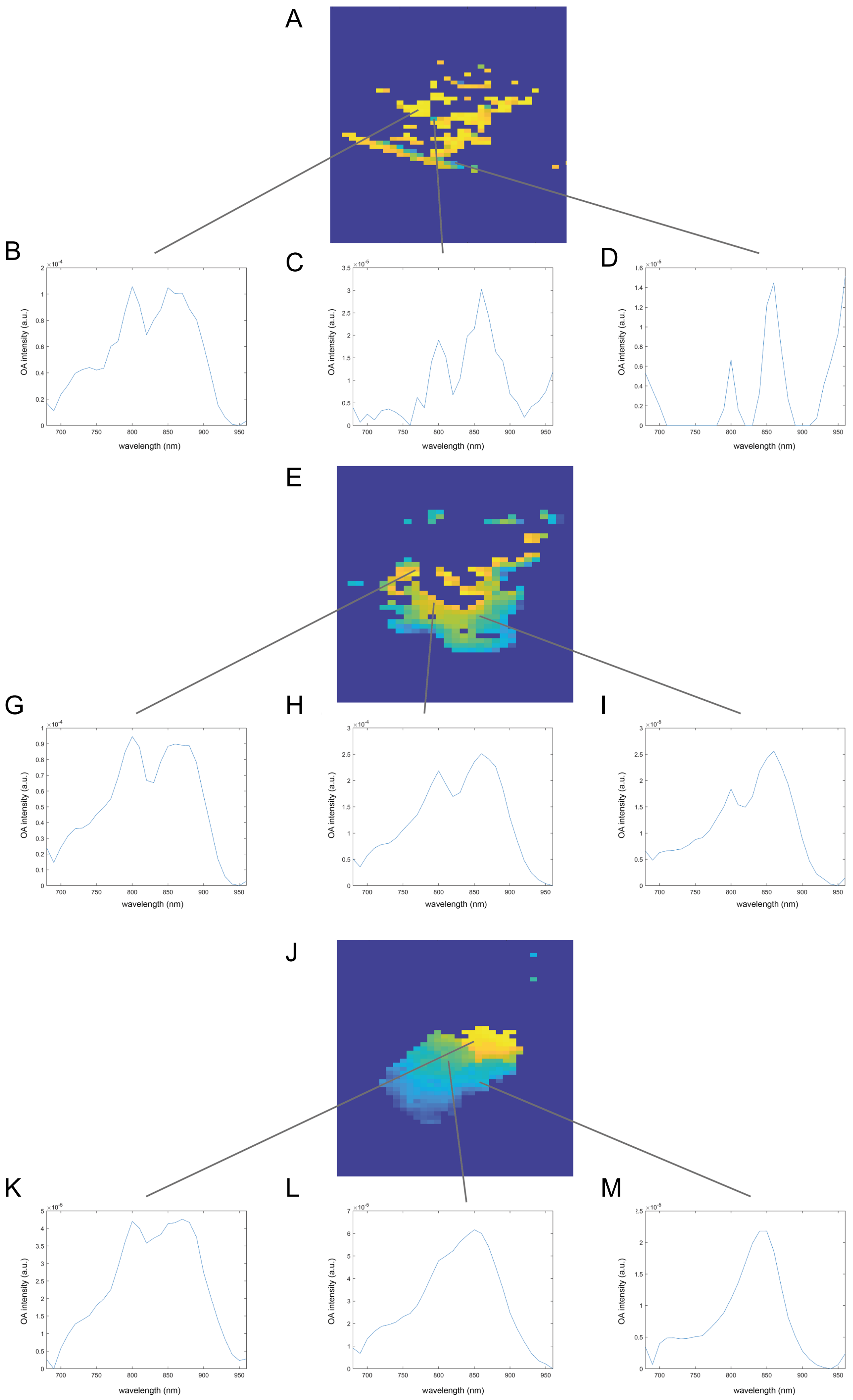

Supplementary Figure 16: Spectra of individual pixels. Shown are selected pixel spectra for the same mice at three different time points (A-D: 0 h, E-I: 24 h and J-M: 168 h past injection), with one slice shown per time point.

.

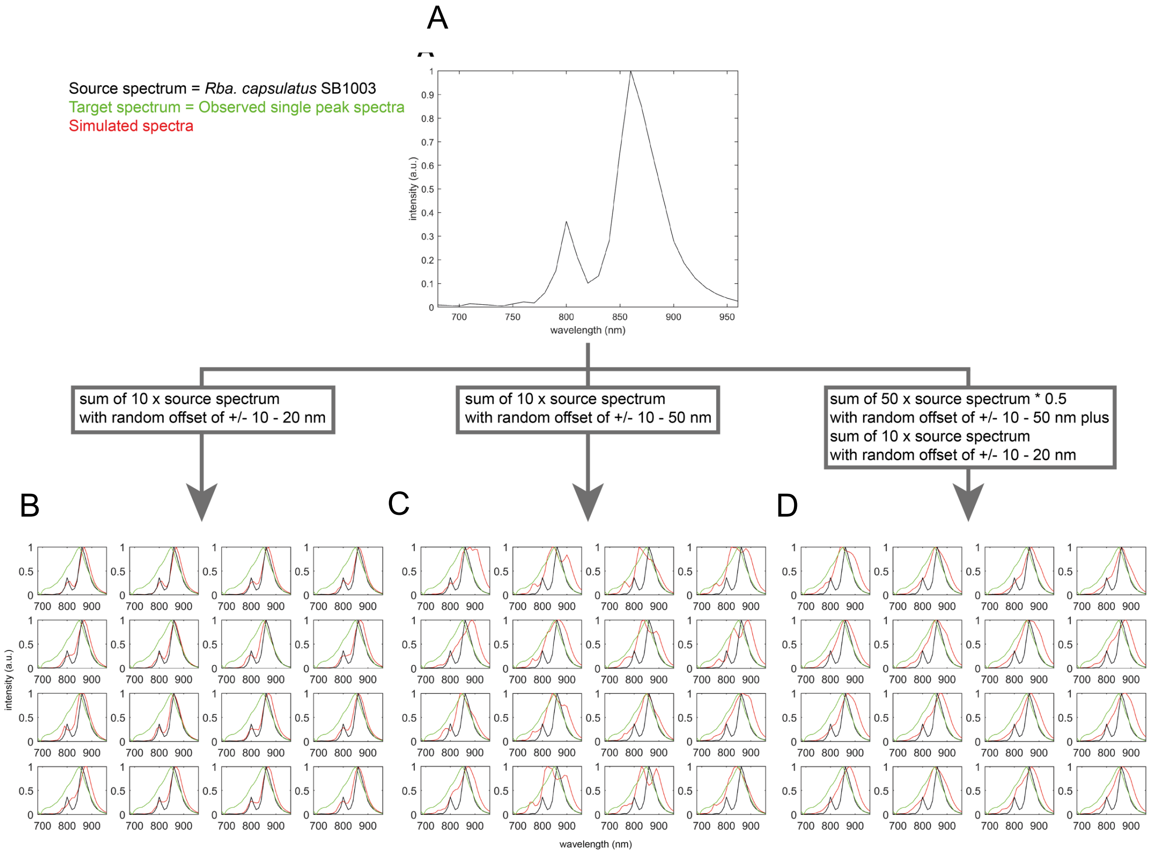

Supplementary Figure 17: Simulated self-convolutions of *Rhodobacter* peaks with spectral uncertainty. For the simulation spectra of *Rba. capsulatus* SB1003 (**A**) have been summed while each spectrum was randomly shifted by +/- 0-20 nm (**B**), by +/- 0-50 nm (**C**) or by a combination of both (**D**). For each case 16 simulated spectra are shown as examples.

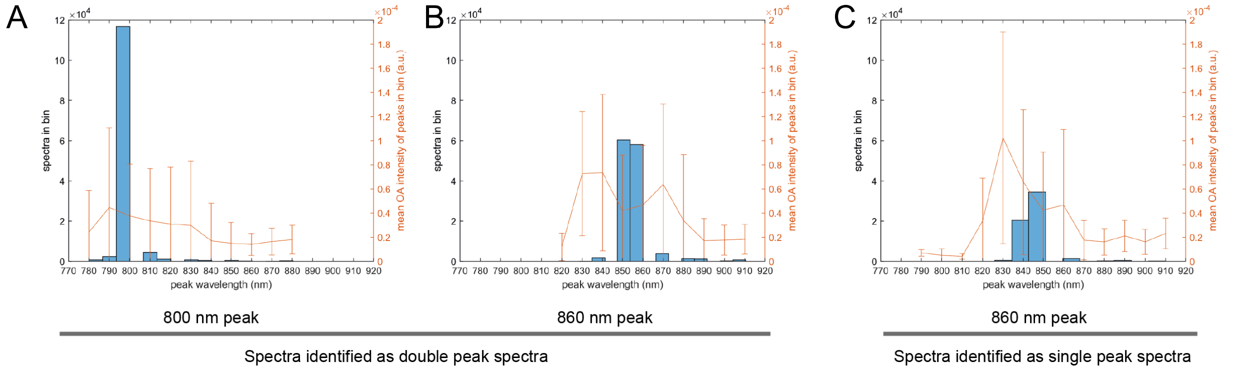

Supplementary Figure 18: Distribution of peak wavelength. Shown for the observed peak types (800/860 nm double peak (A and B and 860 nm single peak (C)). Shown is the number of observed spectra with the given peak wavelength as histogram as well as the mean OA peak intensity of the spectra in the bin.
